# P046: Validation of a simple tool to save resources with previous MRSA carriers

**DOI:** 10.1186/2047-2994-2-S1-P46

**Published:** 2013-06-20

**Authors:** A Agostinho, V Sauvan, L Pagani, P Hoffmeyer, S Harbarth, I Uçkay

**Affiliations:** 1 Infection Control Program, Geneva University Hospitals, Geneva, Switzerland; 2 Orthopaedic Surgery, Geneva University Hospitals, Geneva, Switzerland

## Introduction

According to international recommendations, patients who previously carried Methicilin Resistant *Staphylococcus aureus* (MRSA) should benefit from isolation measures while waiting for MRSA screening results. All these measures are resource consuming. The use of an algorithm, valid and reproducible, for assessing the probability of MRSA carriage on admission may allow better allocation of resources and economies.

## Methods

Cohort study, bi-centric, open from 2010 to 2012, determining the carrying MRSA based on previous carrier states known.

Phase I (2009-10): Determination of the tool [Department of Orthopaedics, University Hospitals of Geneva (HUG)]

Phase II (2012): Evaluation of the reproducibility of the tool and extension of its applicability to other departments of HUG: orthopaedics, neurosurgery, ophthalmology and oto-rhino-laryngology as well as in geriatric care [3-Chêne Hospital].

Inclusion criteria: all patients known to have been MRSA carriers.

Exclusion criteria: all patients not meeting the inclusion criteria.

## Results

### Phase I

43/189 patients (23%) known to have been MRSA carriers had a positive screening on admission. Of these:

- 34 patients (79%) had the last positive result from < 1 year,

- 6 patients (14%) had the last positive screning between 1-2 years,

- 3 patients (7%) had the last positive result from > 2 years.

### Phase II

The validity of the algorithm was tested from January to December 2012. According to the inclusion criteria, 291 acute care and 239 geriatric patients were included.

Considering as only predictive tool of MRSA carriage state the gap of less than 2 years between the last MRSA positive carrier state and admission, there were:

- 68% concordance in the application of this rule (358/530),

- 31% mismatch (164/530), of which:

- 93% (153/164) were negative when a positive test had been predicted,

- 7% (11/164) were MRSA carriers when a negative test had been predicted,

- In 1% of patients (8/530) the algorithm could not be applied due to lack of samples.

## Conclusion

The exclusive use of this algorithm to predict MRSA carrier state is a very simple and reproducible tool, which allows considerable savings with the reduction in the number of days of isolation.

## Disclosure of interest

None declared

